# Esophageal Balloon-Directed Ventilator Management for Postpneumonectomy Acute Respiratory Distress Syndrome

**DOI:** 10.1155/2021/6678080

**Published:** 2021-01-15

**Authors:** Eric Sy, Jagadish Rao, Sherma Zacharias, Juan J. Ronco, James S. Lee

**Affiliations:** ^1^Department of Critical Care, Regina General Hospital, Regina, Saskatchewan, Canada S4P 0W5; ^2^College of Medicine, University of Saskatchewan, Saskatoon, Saskatchewan, Canada S7N 5E5; ^3^Division of Critical Care Medicine, Department of Medicine, Faculty of Medicine, University of British Columbia, Vancouver, British Columbia, Canada V5Z 1M9

## Abstract

**Objective:**

Postpneumonectomy patients may develop acute respiratory distress syndrome (ARDS). There is a paucity of data regarding the optimal management of mechanical ventilation for postpneumonectomy patients. Esophageal balloon pressure monitoring has been used in traditional ARDS patients to set positive end-expiratory pressure (PEEP) and minimize transpulmonary driving pressure (Δ*P*_L_), but its clinical use has not been previously described nor validated in postpneumonectomy patients. The primary objective of this report was to describe the potential clinical application of esophageal pressure monitoring to manage the postpneumonectomy patient with ARDS.

**Design:**

Case report. *Setting*. Surgical intensive care unit (ICU) of a university-affiliated teaching hospital. *Patient*. A 28-year-old patient was involved in a motor vehicle collision, with a right main bronchus injury, that required a right-sided pneumonectomy to stabilize his condition. In the perioperative phase, they subsequently developed ventilator-associated pneumonia, significant cumulative positive fluid balance, and ARDS. *Interventions*. Prone positioning and neuromuscular blockade were initiated. An esophageal balloon was inserted to direct ventilator management. *Measurements and Main Results*. *V*_T_ was kept around 3.6 mL/kg PBW, Δ*P*_L_ at ≤14 cm H_2_O, and plateau pressure at ≤30 cm H_2_O. Lung compliance was measured to be 37 mL/cm H_2_O. PEEP was optimized to maintain end-inspiratory transpulmonary pressure (*P*_L_) < 15 cm H_2_O, and end-expiratory *P*_L_ between 0 and 5 cm H_2_O. The maximal Δ*P*_L_ was measured to be 11 cm H_2_O during the care of this patient. The patient improved with esophageal balloon-directed ventilator management and was eventually liberated from mechanical ventilation.

**Conclusions:**

The optimal targets for *V*_T_ remain unknown in the postpneumonectomy patient. However, postpneumonectomy patients with ARDS may potentially benefit from very low *V*_T_ and optimization of PEEP. We demonstrate the application of esophageal balloon pressure monitoring that clinicians could potentially use to limit injurious ventilation and improve outcomes in postpneumonectomy patients with ARDS. However, esophageal balloon pressure monitoring has not been extensively validated in this patient population.

## 1. Background

Pneumonectomy may be the only surgical option in certain patients with lung cancer, bronchiectasis, pulmonary hemorrhage, or trauma [1]. Following pneumonectomy, approximately 5-10% of these patients may develop acute respiratory distress syndrome (ARDS) during the perioperative phase [2, 3]. Prior studies have described the risk of developing ARDS to be highest with right-sided pneumonectomy, multiple patient comorbidities, large positive fluid balances, and higher tidal volumes during one-lung ventilation [3–5].

According to several international critical care societies, the management of traditional patients with severe ARDS includes the use of low tidal volume (*V*_T_) ventilation at 4-8 mL/kg predicted body weight (PBW) to prevent ventilator-induced lung injury (VILI), prone positioning, neuromuscular blockade, and venovenous extracorporeal membrane oxygenation (VV-ECMO) [6, 7]. However, the optimal ventilation strategy in postpneumonectomy ARDS remains unknown and there remains a paucity of data in this clinical setting. Mortality with postpneumonectomy ARDS is quite high, with observational studies describing mortality ranging from 30 to 80% [2, 4, 8]. Strategies to manage postpneumonectomy ARDS have included the use of low *V*_T_ ventilation, potentially as low as 3 mL/kg PBW in some case reports; however, the ideal settings for positive end-expiratory pressure (PEEP) and *V*_T_ remain unknown [8–10].

Esophageal balloon pressure monitoring has been used by clinicians to guide mechanical ventilation in conventional ARDS to set PEEP, avoid VILI, and minimize transpulmonary driving pressure (Δ*P*_L_). However, its clinical use in postpneumonectomy patients has not been reported. In this case report, we describe the development of ARDS following pneumonectomy and subsequent ventilator management with additional guidance from an esophageal balloon. The primary objective of this study was to describe the potential clinical application for the use of esophageal balloon pressure monitoring in postpneumonectomy patients.

## 2. Case Presentation

A 28-year-old man (height 194 cm, PBW 88 kg) with no previous medical conditions was involved in a single-vehicle collision resulting in ejection from the vehicle. His initial Glasgow coma scale (GCS) score was 12 when the paramedics arrived, which later deteriorated to 3 upon arrival to the emergency department at a tertiary care trauma centre. On physical examination, he had reduced air entry to the right chest with bruising, subcutaneous emphysema, and significant hypoxemia on a nonrebreather mask. On point-of-care ultrasound, the absence of lung sliding was identified over the right chest. Due to significant hypoxemia, he was intubated. A chest tube was inserted into the right pleural space, and a second chest tube was inserted on the right due to an ongoing air leak.

Hypoxemia persisted with a large air leak on the right and substantial difficulties with mechanical ventilation with low return in *V*_T_. A significant bronchopleural fistula or tracheobronchial injury was suspected. Following selective isolation of the right lung with a double-lumen endotracheal tube, the patient's condition stabilized enough to perform a computed tomography (CT) scan. The CT scan confirmed the presence of a large right-sided pneumothorax, extensive pneumomediastinum, right-sided pulmonary contusions, subcutaneous emphysema, and numerous bilateral rib fractures. Other injuries included a stable T4 and an unstable T5 vertebral body fracture, a small 6 mm right subdural hemorrhage, and a left adrenal gland injury. Postadmission, severe acute respiratory syndrome coronavirus 2 (SARS-CoV-2) testing was also performed and was negative. His clinical parameters and ventilator settings over the course of his intensive care unit (ICU) stay are summarized in Table 1.

**Figure 1:**
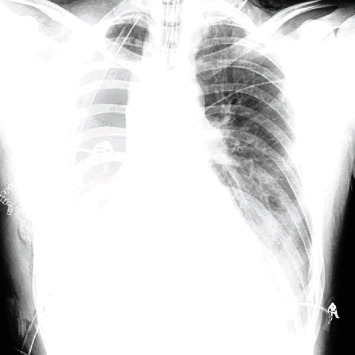
Chest radiograph on post-operative day #1.

The patient was taken to the operating room and diagnosed with a ruptured right main bronchus after an intraoperative bronchoscopy. A right posterior lateral thoracotomy was performed with an initial attempt to repair this injury, but due to ongoing patient instability, a right sleeve pneumonectomy was performed. At the end of the case, the double-lumen endotracheal tube was changed to a single lumen tube, and the patient was transferred to the ICU. A follow-up CT scan of the head demonstrated reduction in the size of the subdural hemorrhage to 3 mm, and follow-up chest radiographs demonstrated evolving atelectasis and opacification in the left lower lobe (Figure 1).

**Figure 2:**
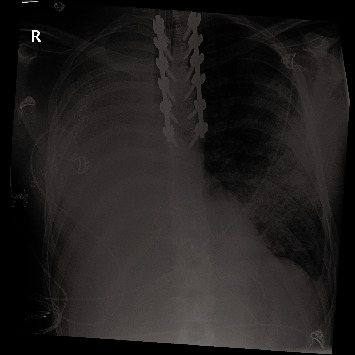
Chest radiograph on post-operative day #11.

On postoperative day (POD) #3, he had T2-T7 posterior instrumentation and fusion. Liberation from the ventilator was attempted; however, the patient could not wean due to secretions and mucous plugging requiring bronchoscopy and tracheobronchial toileting. He was subsequently diagnosed with a *Haemophilus influenzae* ventilator-associated pneumonia (VAP) and started on ceftriaxone on POD #4. By POD #8, the patient had developed worsening left-sided opacities and was eventually diagnosed with ARDS, using the Berlin definition with the exception of bilateral opacities on chest imaging due to the absence of one lung (Figure 2) [11]. A further sputum culture and sensitivity was performed and was positive for *Gardnerella vaginalis*. Subsequently, his antibiotics were changed to piperacillin-tazobactam and moxifloxacin.

He progressed to having worsening hypoxemia and hypercapnia. As a result, he was sedated to a Richmond Agitation-Sedation Scale (RASS) score of -4 to -5. By POD #11, his PaO_2_/FiO_2_ (P/F) ratio had remained in the low 100 s despite sedation, with evidence of patient-ventilator asynchrony. To minimize further VILI, the patient was started on neuromuscular blockade, an adult esophageal balloon (CooperSurgical, Connecticut, United States) was inserted, and prone positioning was initiated. A negative fluid balance was attained with furosemide. After prone positioning and optimization of positive end-expiratory pressure (PEEP), his condition stabilized. His airway driving pressure (Δ*P*) was maintained below 14 cm H_2_O, and esophageal balloon monitoring was used to maintain end-inspiratory transpulmonary pressure (*P*_L_) less than 15 cm H_2_O and end-expiratory *P*_L_ between 0 and 5 cm H_2_O.

The patient further improved and had a tracheostomy on POD #18. He was liberated from the ventilator by POD #25 and decannulated on POD #32. On POD #35, he was discharged home. One month later, he was reviewed in the trauma clinic and he was doing well with ambulation. He had no respiratory concerns and was working on improving muscle strength.

## 3. Discussion

In this study, we report about a patient who developed ARDS following a right pneumonectomy for a traumatic right main bronchus injury. His risk factors for developing ARDS included the presence of VAP, large cumulative positive fluid balance, pulmonary contusions secondary to trauma, and recent right pneumonectomy. During the management of his ARDS with conventional strategies, he developed worsening hypoxemia and patient-ventilator asynchrony, further increasing the risk of patient self-inflicted lung injury (P-SILI). P-SILI may occur due to increased patient respiratory drive and larger *V*_T_, and it may further exacerbate VILI [12]. Consequently, the patient was more deeply sedated, and an esophageal balloon was inserted to monitor ventilatory parameters and guide mechanical ventilation.

Esophageal balloon monitoring has been described for use in patients with ARDS, but it has not been previously described or validated in a clinical setting for postpneumonectomy patients [13, 14]. In ARDS patients, the measured esophageal pressure (*P*_es_) is used as a surrogate for the average pleural pressure (*P*_pl_) when estimating *P*_L_. *P*_L_, or the difference between the airway pressure (*P*_aw_) and *P*_pl_, is represented by the following equation: *P*_L_ = *P*_aw_ − *P*_pl_. However, there are several assumptions for the use of *P*_es_ measurements. Body positioning, heterogeneous lung diseases, and incorrect placement of the esophageal balloon catheter may all impact *P*_es_ measurements [15]. *P*_es_ may be raised in settings of increased abdominal pressure, obesity, or increased intrathoracic edema [16]. Following pneumonectomy, a patient will have a gradual accumulation of fluid in the postpneumonectomy space, as the *P*_pl_ on ipsilateral side equilibrates to zero, and they will consequently develop reduced lung compliance in the remaining lung, as a result of lung hyperinflation [17, 18]. Additionally, shifting of the mediastinum will occur towards the postpneumonectomy space, with raising of the ipsilateral hemidiaphragm [19]. Therefore, it may be unclear what the *P*_es_ represents in the setting of a postpneumonectomy patient with these heterogeneous anatomical changes.

Other heterogeneous lung models may provide insight into the use of *P*_es_ measurements in these circumstances. In anesthetized patients undergoing thoracic surgery during one-lung ventilation, Braunold et al. had described changes with reduced lung compliance despite constant airway pressure, while using esophageal balloon monitoring [20]. In a swine model of unilateral chest wall asymmetry, Cortes-Puentes et al. described the insensitivity of using *P*_L_ to detect global changes within the lungs when a unilateral pleural effusion is present [21]. While a postpneumonectomy patient may not have the same physiology as these examples, these studies illustrate potential limitations of using *P*_es_ as a surrogate for *P*_pl_, in settings of heterogeneous lung or chest wall disease. On the other hand, Pecchiari et al. described that *P*_es_ in rats may still reflect average *P*_pl_ even in mechanically heterogeneous lungs [22]. Although not performed in this case, an occlusion test to measure the ratio of change in airway opening pressure (Δ*P*_es_/Δ*P*_aw_) could be done to validate the use of *P*_es_ measurements further [16]. Analogous changes in Δ*P*_es_/ΔP_aw_ during manual compression of the chest would demonstrate that the *P*_es_ could be used to measure changes in the average *P*_pl_.

In this case, we used *P*_es_ measurements to help supplement our clinical management to minimize lung stress related to elevated Δ*P*_L_. We expected that chest wall elastance would be increased in the setting of a postpneumonectomy patient similar to a patient with a unilateral pleural effusion. However, a postpneumonectomy patient would also likely have more uniform conditions for *P*_es_, unlike a patient with a unilateral pleural effusion where estimates of *P*_es_ are averaged over two lungs. To minimize lung stress, we maintained Δ*P* to less than 14 cm H_2_O. Interestingly, we found that the Δ*P* and Δ*P*_L_ appeared to be correlated, implying that there may be clinical utility in using an esophageal balloon to guide management in postpneumonectomy patients. We also found that this patient's end-expiratory *P*_es_ was elevated, implying a degree of recruitability with higher PEEP in this patient. At a higher PEEP of 18 cm H_2_O, this patient's hypoxemia improved substantially within acceptable ventilatory parameters of *P*_PLAT_, Δ*P*, and Δ*P*_L_. With these manoeuvres, we also maintained a *V*_T_ of approximately 3.6 mL/kg PBW.

However, reduced *V*_T_ may predispose a patient to severe hypercapnia. In a postpneumonectomy patient, alveolar ventilation may occur near dead space, which was an issue that we observed. We addressed instrumental dead space by reducing circuit length and using heated humidification. A prior case report had initiated VV-ECMO and had used tidal volumes as low as 200 mL (approximately 3 mL/kg) [10]. Another case series had initiated VV-ECMO and targeted *V*_T_ to 4 mL/kg PBW with a median *V*_T_ of 277 mL (range 105 to 367 mL) [8]. There was initial consideration of VV-ECMO; however, the patient's condition stabilized without substantial acidosis, after optimization of Δ*P* and PEEP with the additional guidance of *P*_es_ monitoring.

**Figure 3:**
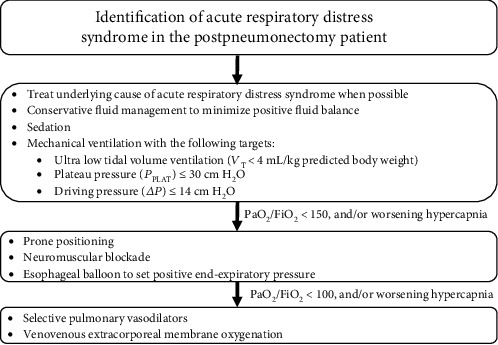
Suggested management approach for post-pneumonectomy patient with acute respiratory distress syndrome.

In summary, a suggested approach for managing the postpneumonectomy ARDS patient could include the following: (a) “ultra” low *V*_T_ aiming <4 mL/kg PBW, (b) conservative fluid management, (c) optimization of sedation with neuromuscular blockade when necessary, and (d) prone positioning (Figure 3). Finally, the use of an esophageal balloon to further optimize PEEP, selective pulmonary vasodilators, and/or VV-ECMO could be considered if the patient remains hypoxemic or develops clinically significant hypercapnia.

## 4. Conclusions

The management of postpneumonectomy ARDS is complex, and the risk of mortality in this perioperative phase is high. Strategies to minimize VILI and P-SILI are important in this phase, although the optimal targets for ventilation and monitoring are unknown. Esophageal balloon monitoring may be an additional tool that clinicians could use in this clinical scenario to limit injurious ventilation, set PEEP, and improve patient outcomes. Further study and validation of esophageal balloon measurements may be warranted in postpneumonectomy patients.

## Figures and Tables

**Table 1:** Patient clinical characteristics and ventilator parameters during his intensive care unit stay.

Patient characteristic	POD #0	POD #5	POD #8	POD #11	POD #12	POD #13	POD #14	POD #16	POD #18
*Clinical parameters*									
Actual weight, kg	87.5	93	99.5	98	98.7	102.9	98.1	96.8	92.6

*Respiratory parameters*									
Ventilator model	PB 840	PB 840	PB 840	PB 840	Hamilton G5	Hamilton G5	Hamilton G5	Hamilton G5	Hamilton G5
Ventilator mode	PCV	PCV	PCV	PCV	APVcmv	APVcmv	APVcmv	APVcmv	APVcmv
Set pressure control	14	18	16	15	-	-	-	-	-
Actual tidal volume, mL	380	425	417	521	410	320	320	346	542
Actual tidal volume, mL/kg PBW	4.3	4.8	4.7	5.9	4.7	3.6	3.6	3.9	6.2
Set respiratory rate, breaths/min	24	26	18	10	24	28	28	22	22
Actual respiratory rate, breaths/min	24	26	19	17	24	28	28	26	26
Minute ventilation, L/min	9.6	10.6	8.7	7.9	9.8	9.0	9.0	9.0	8.9
FiO_2_	0.50	0.50	0.70	0.80	0.80	0.50	0.35	0.40	0.35
pH	7.35	7.36	7.29	7.27	7.37	7.29	7.38	7.44	7.44
PaCO_2_, mm Hg	52	51	75	84	63	71	67	58	52
PaO_2_, mm Hg	130	80	67	82	76	77	85	88	71
HCO_3_, mmol/L	27	28	32	34	35	31	37	37	35
PaO_2_ : FiO_2_, mm Hg	260	160	96	103	95	154	243	220	203

Airway pressure, cm H_2_O									
Plateau	-	-	-	-	28	28	26	16	-
Peak	23	29	32	32	35	32	28	23	17
Mean	14	17	20	21	21	23	22	15	15
Set PEEP, cm H_2_O	8	10	14	14	14	18	18	12	12
Intrinsic PEEP, cm H_2_O	-	-	-	-	1	0	0	0	-
*P*_es_, cm H_2_O	-	-	-	-					
At end-inspiration	-	-	-	-	17	18	14	-	-
At end-expiration	-	-	-	-	15	17	13	-	-
*P*_L_, cm H_2_O	-	-	-	-					
At end-inspiration	-	-	-	-	11	10	12	-	-
At end-expiration	-	-	-	-	0	1	5	-	-
Airway driving pressure, cm H_2_O	-	-	-	-	13	10	8	4	-
Transpulmonary driving pressure, cm H_2_O	-	-	-	-	11	9	7	-	-
Respiratory system compliance, mL/cm H_2_O	-	-	-	-	32	32	40	87	-
Lung compliance, mL/cm H_2_O	-	-	-	-	37	36	46	-	-
Chest wall compliance, mL/cm H_2_O	-	-	-	-	205	320	320	-	-

*Cointerventions*									
Prone positioning	No	No	No	No	Yes	Yes	Yes	No	No
Neuromuscular blockade	No	No	No	No	Yes	Yes	Yes	No	No

Abbreviations: APVcmv: adaptive pressure ventilation continuous mandatory ventilation; PB: Puritan Bennett; PCV: pressure-controlled ventilation; *P*_es_: esophageal pressure; *P*_L_: transpulmonary pressure; POD: post-operative day; -: not measured or recorded in the chart.

## Abbreviations

Δ*P*: Airway driving pressure
Δ*P*_L_: Transpulmonary driving pressure
ARDS: Acute respiratory distress syndrome
CT: Computed tomography
CXR: Chest radiograph
ECMO: Extracorporeal membrane oxygenation
GCS: Glasgow coma scale
ICU: Intensive care unit
P/F: PaO_2_/FiO_2_ ratio
PEEP: Positive end-expiratory pressure
*P*_aw_: Airway pressure
*P*_es_: Esophageal pressure
*P*_L_: Transpulmonary pressure
*P*_pl_: Pleural pressure
*P*_PLAT_: Plateau pressure
P-SILI: Patient self-induced lung injury
VAP: Ventilator-associated pneumonia
VILI: Ventilator-induced lung injury
*V*_T_: Tidal volume.
